# *In ovo* CpG DNA delivery increases innate and adaptive immune cells in respiratory, gastrointestinal and immune systems post-hatch correlating with lower infectious laryngotracheitis virus infection

**DOI:** 10.1371/journal.pone.0193964

**Published:** 2018-03-07

**Authors:** Mohamed Sarjoon Abdul-Cader, Aruna Amarasinghe, Victor Palomino-Tapia, Hanaa Ahmed-Hassan, Khawaja Bakhtawar, Eva Nagy, Shayan Sharif, Susantha Gomis, Mohamed Faizal Abdul-Careem

**Affiliations:** 1 Health Research Innovation Center, Department of Ecosystem and Public Health, Faculty of Veterinary Medicine, University of Calgary, Calgary, Alberta, Canada; 2 Zoonoses Department, Faculty of Veterinary Medicine, Cairo University, Giza, Egypt; 3 Department of Pathobiology, University of Guelph, Guelph, Ontario, Canada; 4 Department of Veterinary Pathology, Western College of Veterinary Medicine, University of Saskatchewan, Saskatoon, Saskatchewan, Canada; The University of Melbourne, AUSTRALIA

## Abstract

Cytosine-guanosine deoxynucleotides (CpG) DNA can be delivered *in ovo* at embryo day (ED)18 for the stimulation of toll-like receptor (TLR)21 signaling pathway that ultimately protects chickens against a number of bacterial and viral infections. There is a dearth of information understanding the mechanisms of protection induced by *in ovo* delivered CpG DNA. The objective of this study was to determine the immune cell changes post-hatch following *in ovo* delivery of the TLR21 ligand, CpG DNA. In order to quantify changes of percentage of KUL01+, IgM+ B, cluster of differentiation (CD)4+ and CD8α+ cells, trachea, lung, duodenum, large intestine, spleen and bursa of Fabricius were collected on day 1 post-hatch. We found increased recruitments of KUL01+ cells, in organs of these body systems post-hatch following *in ovo* delivery of CpG DNA. Although IgM+ B cells, CD4+ and CD8α+ cells were increased in lungs and immune system organs, these cells were not quantifiable from the trachea, duodenum and large intestine immediately following the hatch. Furthermore, when CpG DNA is delivered *in ovo* and subsequently infected with infectious laryngotracheitis virus (ILTV) post-hatch on day 1, CpG DNA reduces morbidity and mortality resulting from ILTV infection. This study provides insights into the mechanisms of host responses elicited following *in ovo* delivery of CpG DNA in avian species.

## Introduction

CpG DNA is classified into three major classes, class A, B and C based on the structural variations and their effects on peripheral blood mononuclear cell (PBMC)s [1, 2]. Class A CpG DNA mainly activates the dendritic cell (DC)s and natural killer (NK) cells mediated *via* interferon regulatory factor (IRF)7 signaling pathways from early endosomes leading to increasing production of type 1 interferon (IFN)s. The class B CpG DNA is a strong activator of B cells and monocytes and operates *via* nuclear factor (NF)-kB signaling pathway from late endosomes leading to the production of pro-inflammatory mediators. Class C CpG DNA shows the characteristics of both class A and B [3, 4] in terms of the structure and functions. Toll-like receptor (TLR)9 in mammals and TLR21 in avian species detect both bacterial and viral DNA containing unmethylated CpG motifs [5], which are generally methylated in the genomes of vertebrate [6, 7]. The frequency of CpG motifs is also negligible in vertebrate DNA, while it occurs with high frequency in microbial genomes [4] and that allow elicitation of host responses against DNA of microbial origin and not against the host origin.

Induction of innate host responses by the treatment of CpG DNA has been studied in various animal models. For example, many studies in the mouse model reported that treatment of CpG DNA significantly stimulates the recruitment of innate immune cells such as macrophages and NK cells in the respiratory and genital mucosal epithelium [8, 9] correlating with the inhibition of viral replication in the subsequent challenges with herpes simplex virus (HSV)-2 [8] and influenza virus [10] respectively. CpG DNA is also known to increase adaptive immune cells such as B cells and T cell subsets *via* increased cell proliferation and cell survival, which has been recorded in mammals [11–14]. In avian species, there is an indication that CpG DNA induce proliferation of B cells *in vitro* [15] and B cells and T cell subsets *in vivo* in four weeks old chickens [16].

Pre-hatch or *in ovo* vaccination is a major advancement in infectious disease control in chickens and it is practiced at embryo day (ED) 18. When the eggs are hatched three days following the *in ovo* vaccination and placed the newly hatched chickens in poultry barns, a number of vaccines have been introduced to the chicks reducing the window of susceptibility for various infectious diseases [17]. *In ovo* delivered CpG DNA has been shown to reduce microbial infections encountered post-hatch in chickens such as bacterial infections [18–20] and viral infections [9, 21] correlating with macrophage response in lungs. However, it is not known whether *in ovo* delivered CpG DNA is capable of eliciting 1) macrophage responses post-hatch in other body systems and 2) adaptive immune cells in respiratory and other body systems. In the present study, we investigated whether the prophylactic use of *in ovo* delivered TLR21 ligand, CpG DNA could stimulate mucosal immune responses in lungs, trachea, duodenum, large intestine, spleen and bursa of Fabricius post-hatch potentially reducing infection of infectious laryngotracheitis virus (ILTV). Our data demonstrate that *in ovo* delivery of CpG DNA increases recruitments of KUL01+, IgM+ B cells, CD4+ and CD8α+ cells day 1 post-hatch at variable extents. When the chickens were infected with ILTV at day 1 of age coinciding with this augmented cellular response induced by *in ovo* delivered CpG DNA, the ILTV induced morbidity and mortality were reduced potentially minimizing the replication of the virus indicating that *in ovo* delivery CpG DNA may be a prophylactic measure against ILTV infection.

## Materials and methods

### Animals

The Veterinary Science Animal Care Committee (VSACC) and Health Science Animal Care Committee (HSACC) have approved the use of SPF eggs, embryos, and chickens used in all our experimental procedures (animal Protocol #: AC13-0291). The sampling of chicken tissues was performed as has been approved by the institutional animal care committees. Briefly, for embryo sampling at the ED19, the egg shell was cut opened at the broader end of the egg, fetal membranes were disrupted and the embryos were decapitated. For the sampling of tissues post-hatch at day 1 and at endpoints, the chickens were euthanized using overdose of isoflurane anesthesia followed by cervical dislocation. The eggs purchased from Canadian Food Inspection Agency (CFIA), Ottawa, Canada were incubated at Health Research Innovation Center (HRIC), University of Calgary in digital incubators (Rcom Pro 20 and 50, Kingsuromax 20 and Rcom MARU Deluxe max, Autoelex Co., Ltd., GimHae, GyeongNam, Korea). During the incubation (60–70% relative humidity and 37.2–37.6°C temperature depending on the stage of the incubation) [22], the eggs were candled to select fertile eggs at ED11 for the experiments.

### Virus and TLR ligand

The ILTV (strain N-71851) purchased from the American Type Culture Collection (ATCC, Manassas, Virginia, United States), was used in the studies [9, 23]. Initially, the viral titer was determined by plaque assay done using leghorn chicken hepatocarcinoma (LMH) cells [24].

The ligand for TLR21, Class B CpG DNA 2007 (5'-TCG TCG TTG TCG TTT TGT CGT T-3'), and non-CpG DNA (5'-TGC TGC TTG TGC TTT TGT GCT T-3') (Invivogen, San Diego, California, USA) were purchased from Cedarlane (Burlington, ON, Canada). These class B CpG DNA sequences, which consist of a complete phosphorothioate backbone, have been shown to induce strong monocyte and B cell responses when compared to non-CpG DNA [25, 26].

### Experimental design

#### Characterization of cell-mediated immune responses in the embryo (ED19) lungs following *in ovo* delivery CpG DNA

In order to determine whether *in ovo* delivery of CpG DNA leads to cellular responses characterized by KUL01+, IgM+ B, CD4+ and CD8α+ cells, the lung tissues that originated from in *ovo* delivered CpG DNA (50 μg CpG DNA in 200 μL PBS per egg) group (*n* = 9), control group injected with 50 μg non-CPG DNA in 200 μL PBS *in ovo* (*n* = 4) and *in ovo* PBS treated group (n = 6) were collected and embedded in OCT compound (Tissue-Tek^®^, Sakura Finetek USA inc, Torrance, CA, USA) 24 hours following the *in ovo* treatments. The OCT blocks were snap frozen in dry ice and stored at -80°C before being sectioned.

#### Characterization of cell-mediated immune responses in multiple body systems of day 1 aged chickens following *in ovo* delivery CpG DNA

To study the potential cellular mediators of antimicrobial response, such as KUL01+, IgM+ B, CD4+ and CD8α+ cells elicited by CpG DNA, we delivered CpG DNA (50μg of CpG DNA diluted with 200μl of sterile PBS per egg, n = 5) *in ovo* at ED18 in SPF chicken eggs, while the two control groups received either 50 μg non-CPG DNA in 200 μL PBS *in ovo* (*n* = 4) or 200μl of sterile PBS per egg *in ovo* (n = 4). At day 1 post-hatch, the lungs, tracheas, duodenum, large intestine, spleen, and bursa of Fabricius were collected and preserved in OCT compound (Tissue-Tek^®^, Sakura Finetek USA inc, Torrance, CA, USA), snap frozen in dry ice and stored at -80°C before being sectioned.

#### Determination of antiviral response elicited by *in ovo* delivered CpG DNA against ILTV infection encountered post-hatch

To investigate the antiviral response elicited by *in ovo* delivered CpG DNA in lungs, trachea, duodenum, large intestine, spleen and bursa of Fabricius against post-hatch ILTV infection, ED18 SPF eggs were delivered *in ovo* either with 50μg of CpG DNA (diluted with 200μl of sterile PBS per egg, n = 8) or 50μg of non-CpG DNA in 200μl of sterile PBS per egg (n = 7). Both groups were infected with ILTV intra-tracheally at day 1 post-hatch (3x10^4^ plaque forming units or PFUs in 30μl/chicken). To facilitate the intra-tracheal ILTV infection using a pipette, the chicken tongue was gently pulled out and downwards while under isoflurane anesthetic, exposing the glottis, the opening that connects to the larynx. Then, the chickens were observed for 12 days and clinical signs and endpoints determined. The clinical signs were scored on a scale of 0–5 (No clinical signs = 0, droopy wings/ ruffled feathers and huddling together/ depression or inactive with lowered head/ loss of body weight/ mild increase of respiration = 1, moderate increase of respiration = 2 and severe increase of respiration or gasping = 3). When a birds reached a cumulative score of 5 (humane end point), the chickens were euthanized [9]. At 4 days post-infection, oropharyngeal and cloacal swabs were collected using Puritan^®^ UniTranz-RT^®^ Media Transport Systems (VWR, Edmonton, AB, Canada), which consisted of the sterile swabs and transport medium and ILTV genome quantified following DNA extraction. Since ILTV replicates mainly in tracheal epithelium, we preferred oropharyngeal swabs rather than tracheal swabs of 5 days old chickens in order to prevent any inadvertent injury to tracheal mucosa. When the chickens reached the humane and experimental endpoints, the lungs were collected and preserved in OCT compound (Tissue-Tek^®^, Sakura Finetek USA inc, Torrance, CA, USA), snap frozen in dry ice and stored at -80°C before being sectioned. Additionally, a portion of the lungs also collected in RNA Save (Biological Industries, FroggaBio, Toronto ON, Canada) and stored in -20°C before being used for DNA extraction.

### *In ovo* delivery technique

Briefly, the egg shell was punctured through the air sac at the broader end of the eggs using a sterile 23-gauge needle after disinfecting the shell surface with 70% ethanol. Two and a half centimeters long, a 23-gauge needle was then used for *in ovo* delivery of compounds into the amniotic cavity by inserting the entire length of the needle perpendicularly through the punctured hole. At the end of the procedure, the holes were sealed with lacquer and eggs were placed in the incubator for further incubation.

### Immunofluorescent assay

The tissues preserved in OCT were sectioned (thickness of 5 micrometer, μm) and indirect immunofluorescent assay was used to quantify KUL01+, IgM+ B cells, CD4+cells and CD8α+ cells numbers in lungs, trachea, duodenum, large intestine, spleen and bursa of Fabricius. For KUL01+, IgM + B cell, CD4+ cell and CD8α+ cell staining, 5% goat serum in TBS buffer (Trizma base: 2.42g, NaCl: 8g in 1liter (L) of distilled water, pH 7.6) was used for the purpose of blocking and incubated at room temperature for 30 minutes in a humidified chamber. Unlabeled mouse monoclonal antibody specific for chicken macrophages/monocytes/interdigitating cells, KUL01 (Southern Biotech, Birmingham, Alabama, USA), IgM (M-4, Southern Biotech, Birmingham, Alabama, USA), CD4 (CT-4, Southern Biotech, Birmingham, Alabama, USA) and CD8α (CT-8, Southern Biotech, Birmingham, Alabama, USA) were used in 1:200 dilution (except IgM which was used at 1:100 dilution) in blocking buffer and incubated for 30 minutes at the room temperature in a humidified chamber. For KUL01+, IgM and CD8α+ cell staining DyLight^®^ 550 conjugated goat anti-mouse IgG (H+L) (Bethyl Laboratories Inc., Montgomery, TX, USA) was used in 1:500 dilution in blocking buffer as the secondary antibody and incubated for 1 hour at the room temperature in a humidified chamber. For CD4+ cell staining biotinylated Goat Anti-Mouse IgG (H+L) (Southern Biotech, Birmingham, Alabama, USA) was used in 1:250 dilution in blocking buffer and incubated for 30 minutes and then incubated with DyLight^®^ 488 streptavidin in 15:1000 dilution for 30 minutes at the room temperature in a humidified chamber. TBS-T buffer (TBS with 0.1% Tween 20) was used as the washing buffer followed by PBS and after each step, the sections were washed 3 times (twice with TBS-T buffer and once with PBS) with an interval of 3–5 mins. Finally, the slides were mounted in Vectashield mounting medium with 4′, 6-Diamidine-2′-phenylindole dihydrochloride (DAPI) (Vector Laboratories Inc., Burlingame, CA, USA), cover slipped and edges sealed with lacquer.

### DNA extraction and real time polymerase chain reaction (PCR)

From the swab samples collected at 4 days post-infection and also lungs collected from chickens that reached the end points, DNA extraction was carried out using QIAamp DNA mini kit (QIAGEN GmbH, Hilden, Germany) as per manufacturer’s guidelines and the concentration of DNA was quantified using the Nanodrop 1000 spectrophotometer at the wavelength of 260/280 nm (ThermoScientific, Wilmington, DE, USA). Two hundred nano grams (ng) (cloacal swabs, lungs) or 25 ng (oropharyngeal swabs) of the extracted DNA were used for real-time PCR. The real time PCR assay was conducted in a 96-well PCR plate (VWR, Edmonton, AB, Canada) in duplicate to quantify the protein kinase (PK) gene of ILTV in relation to β actin housekeeping gene. Briefly, the calculated copy numbers of PK gene were normalized with the copy numbers of β-actin gene and expressed for 1 x 10^6^ host cells). In order to generate a standard curve, a dilution series of ILTV plasmids (cloned into the pCR^®^2.1-TOPO^®^ vector and amplified in One Shot^®^ *Escherichia coli*, TOPO TA Cloning kit Top 10 (Invitrogen, Burlington, ON, Canada) were used in duplicate with copy numbers ranging from 1.56 x 10^3^-1.56x10^10^. Fast SYBR^®^ Green Master Mix (Invitrogen, Burlington, ON, Canada) was used in 20μl of reaction volume. The detection of intercalating SYBR^®^ Green dye was conducted in a Thermal Cycler (CFX96-C1000) (Bio-Rad Laboratories, Mississauga, ON, Canada). Five pico molar (pM) of ILTV PK (ORF2) gene specific primers (F: 5’-TAC GAT GAA GCG TTC GAC TG -3’ and R: 5’-AGG CGT GAC AGT TCC AAA GT -3’) [27, 28] or β actin primers (F: 5’-CAA CAC AGT GCT GTC TGG TGG TA-3’ and R: 5’-ATC GTA CTC CTG CTT GCT GAT CC -3’) [9, 29] were used in each reaction and gene specific plasmids were included as a positive control and DNAse/RNAse free water was included as a negative control. The optimum parameters for thermal cycling were 95°C for 20 seconds (s) of pre-incubation, 95°C for 3s of 40 amplification cycles with the final segment of 60°C for 30s. Melting curve was analyzed at 95°C for 10s, 65°C for 5s and finally 9°C for 5s. Acquisition of fluorescent signals was performed at 60°C for 30s.

### Data analyses

For the quantification of numbers of KUL01+, IgM + B cells, CD4+ cells and CD8α+ cells in the examined tissues, 3–5 areas with highest DyLight^®^ 550 (KUL01+, IgM+ B cells and CD8α+ cells) or DyLight^®^ 488 (CD4+ cells) fluorescent signals and corresponding nuclear stained (DAPI) areas were captured under 40X magnification from each section. Then, these images were subjected to fluorescent intensity quantification using the Image-J software (National Institute of Health, Bethesda, Maryland, USA). The resultant fluorescent intensities for DyLight^®^ 550 or DyLight^®^ 488 positive signals were expressed relative to the total areas (as estimated by nuclear staining with DAPI) as a percentage [21].

Student’s *t*-test (GraphPad Prism Software 5, La Jolla, CA, USA) was used for the purpose of identifying the differences between two groups. When more than two groups are parts of an experiment, the one-way analysis of variance (ANOVA) with Bonferroni's post test for selected comparison was performed to identify the differences between groups. In addition, Log-rank test was used to identify group differences in survival percentage and Mann-Whitney U test was used to identify group differences in clinical score data for selected time points. Before being analyzed each set of data, the outlier test was conducted using the Grubbs’ test (GraphPad software Inc., La Jolla, CA, USA). The differences between groups were considered statistically significant at P ≤ 0.05.

## Results

### *In ovo* delivery of CpG DNA increases KUL01+ and CD4+ cells in the lungs pre-hatch while PBS and non-CpG DNA (control CpG DNA) induce similar minimal cellular responses

**Fig 1:**
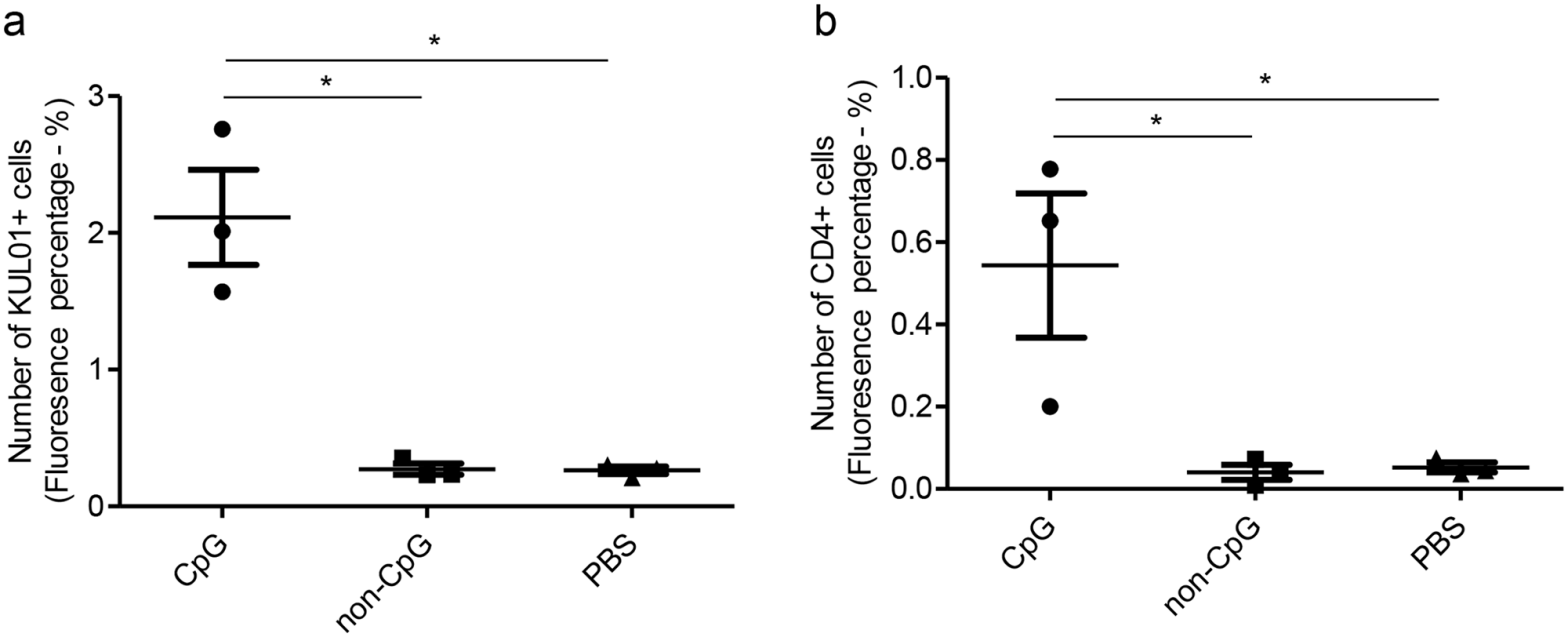
*In ovo* delivery of CpG DNA increases KUL01+ and CD4+ cell numbers in lungs pre-hatch. SPF ED18 eggs were injected with CpG DNA (n = 9), non-CpG DNA (n = 4) or PBS (n = 6). At ED19, lungs were collected and immunofluorescent assay was performed for KUL01+ and CD4+ cells. The quantitative data for KUL01+ (a) and CD4+ cells (b) are shown. The one-way ANOVA test with Bonferroni's post test for selected comparison was performed to identify group differences and the differences were considered significant at P< 0.05. The bars represent mean ± SEM.

We examined pre-hatch lungs for KUL01+, IgM + B cells and CD4+ and CD8α+ cell subsets following *in ovo* delivery of CpG DNA, non-CpG DNA and PBS. We were able to detect and quantify only KUL01+, and CD4+ cells (Table 1). We found that *in ovo* delivered CpG DNA at ED18 increased the number of KUL01+ (P<0.05) and CD4+ cells (P = 0.02) in pre-hatch lungs (ED19) compared to the controls (Fig 1). The KUL01+ and CD4+ cell responses between non-CpG DNA and PBS groups were similar (P>0.05).

**Table 1:** The cellular responses following *in ovo* delivery of CpG DNA in different organs pre- and post-hatch.

Cell types	Pre-hatch (Lungs)	Post-hatch					
		Lungs	Trachea	Duodenum	Large intestine	Spleen	Bursa of Fabricius
**KUL01+ cells**	√ *	√*	√*	√ *	√ *	√	√ *
**CD4+ cells**	√ *	√ *	-	-	-	√ *	√ *
**CD8α+ cells**	-	√ *	-	-	√	-	√
**IgM+ B cells**	-	√	-	-	-	√ *	-

* indicates significant increase in CpG DNA group at P ≤ 0.05 compared to PBS or non-CpG or both groups

√ indicates an increased trend with CpG DNA treatment and—indicates no changes/not quantifiable.

### *In ovo* delivered CpG DNA increases KUL01+ cells in lungs, trachea, duodenum, large intestine and bursa of Fabricius post-hatch

**Fig 2:**
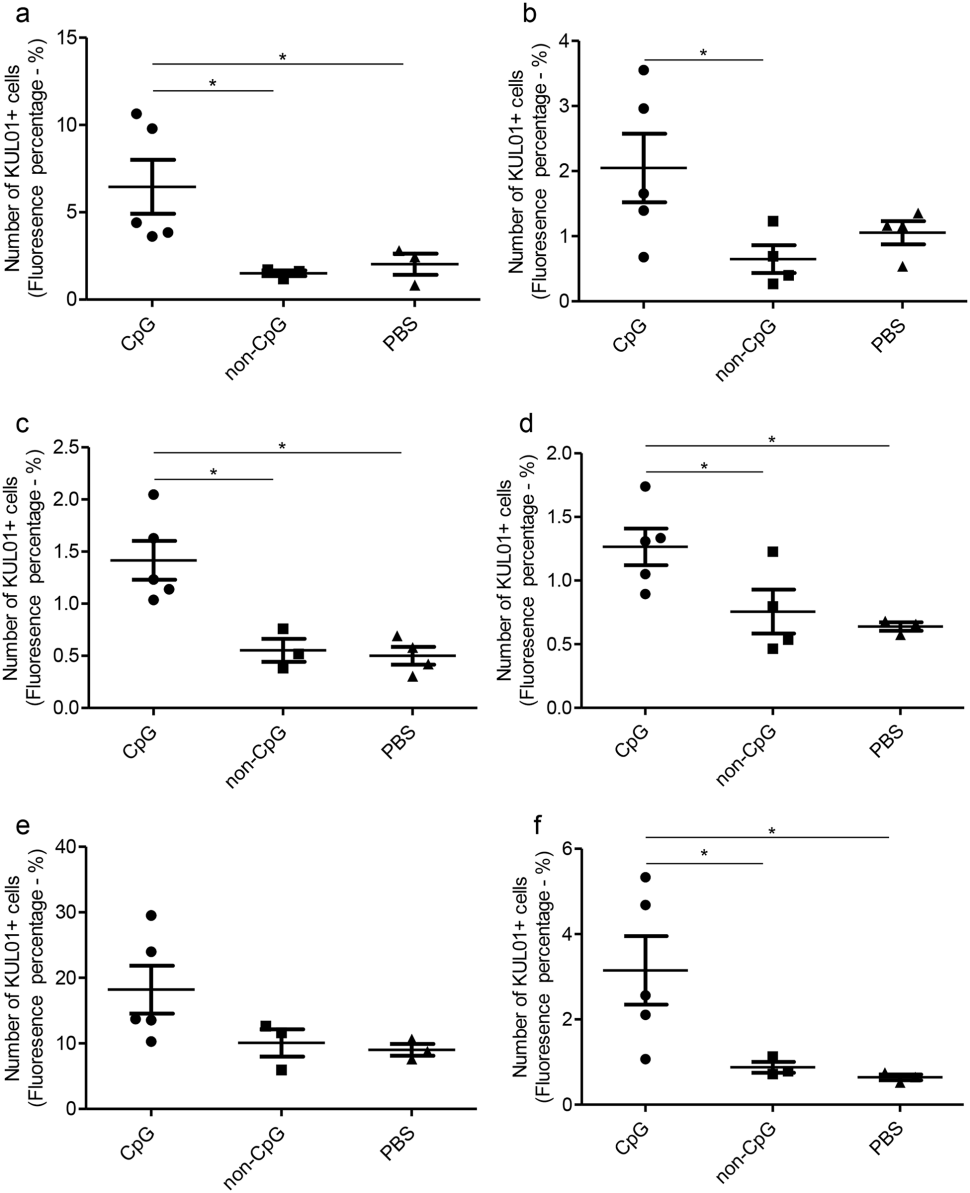
*In ovo* delivered CpG DNA increases KUL01+ cell numbers in lungs, trachea, duodenum, large intestine and bursa of Fabricius. SPF ED18 eggs were delivered with CpG DNA (n = 5), non-CpG DNA (n = 4) or PBS (n = 4) *in ovo* and the eggs were incubated to hatch. At day 1 post-hatch, samples of lungs, trachea, duodenum, large intestine, spleen and bursa of Fabricius were collected in OCT, sectioned and immunofluorescent assay was performed to quantify KUL01+ cells. The quantitative data from the immunofluorescent assay of each organ is shown a) lungs, b) tracheas, c) duodenum, d) large intestine, e) spleen, f) bursa of Fabricius. The one-way ANOVA test with Bonferroni's post test for selected comparison was performed to identify group differences and the differences were considered significant at P< 0.05. The bars represent mean ± SEM.

Since we observed that *in ovo* delivered CpG DNA induces KUL01+and CD4+ cell responses pre-hatch compared to PBS and non-CpG DNA controls in lungs, then we investigated to see whether there is similar cellular response day 1 post-hatch. First, we found that *in ovo* delivered CpG DNA at ED18 increased the number of KUL01+ cells post-hatch in lungs (P<0.05, Fig 2a), trachea (P<0.05, Fig 2b), duodenum (P <0.05, Fig 2c), large intestine (P<0.05, Fig 2d) and bursa of Fabricius (P<0.05, Fig 2f) when compared to non-CpG DNA delivered group. The KUL01+ cell numbers in *in ovo* CpG DNA delivered group was also significantly higher in lungs (P<0.05, Fig 2a), duodenum (P <0.05, Fig 2c), large intestine (P<0.05, Fig 2d) and bursa of Fabricius (P<0.05, Fig 2f) when compared to PBS control group. In the spleen, the differences of KUL01+ cell numbers between *in ovo* CpG DNA delivered and control groups was not significant (P>0.05, Fig 2e). The KUL01+ cell recruitment between non-CpG DNA and PBS control groups were similar (P>0.05, Fig 2). Fig 1 illustrates the KUL01+ cells responses in lungs, trachea, duodenum, large intestine, spleen and bursa of Fabricius post-hatch following *in ovo* CpG DNA, non-CpG DNA or PBS deliveries.

### *In ovo* delivered CpG DNA increases cells of adaptive immune system only in the lungs, spleen and bursa of Fabricius post-hatch

**Fig 3:**
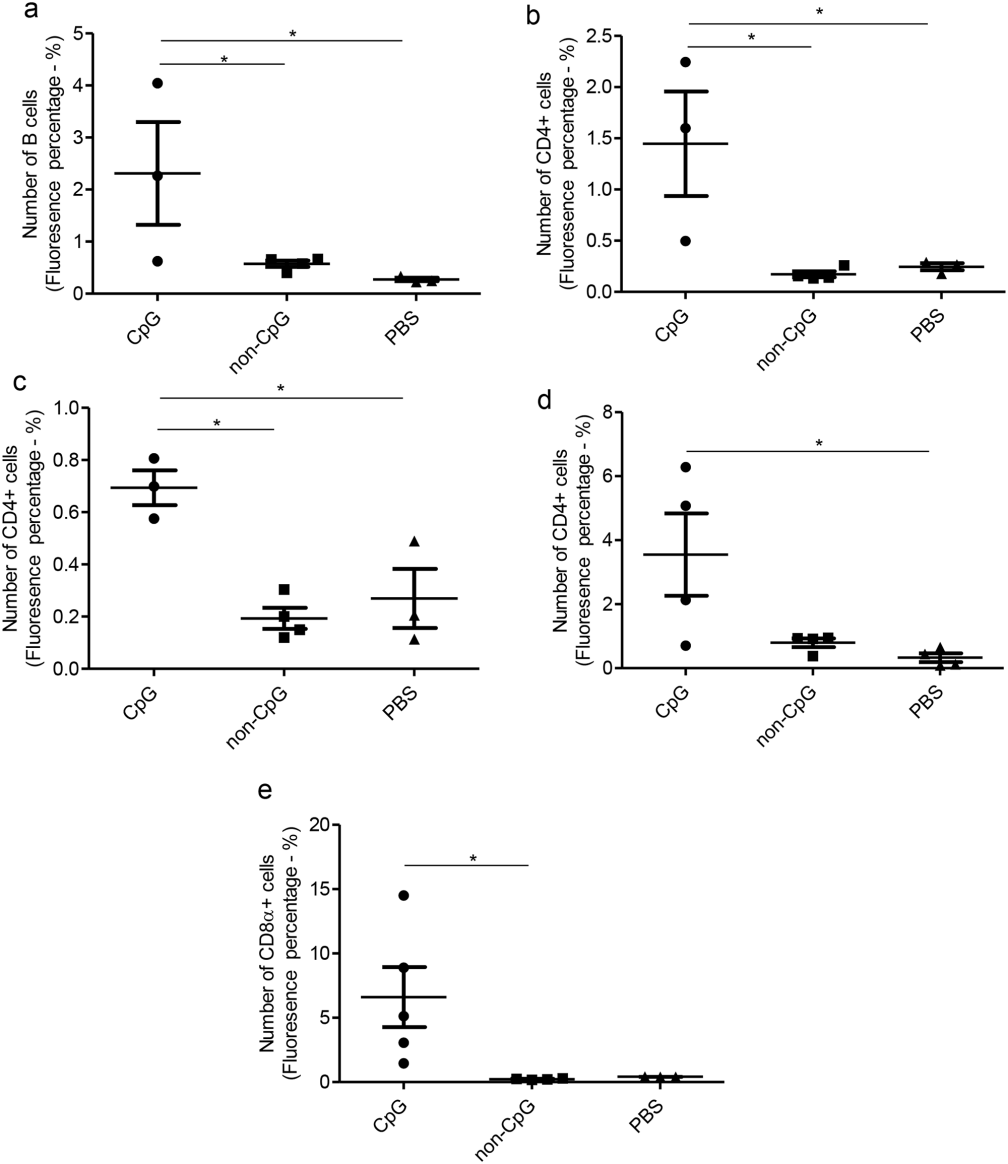
*In ovo* delivery of CpG DNA increases IgM+ B cells in spleen, CD4+ cells in lungs, bursa of Fabricius and spleen and CD8α+ cells in lungs post-hatch. SPF ED18 eggs were delivered with CpG DNA (n = 5), non-CpG DNA (n = 4) or PBS (n = 4) *in ovo* and the eggs were incubated to hatch. At day 1 post-hatch, lungs, trachea, duodenum, large intestine, spleen and bursa of Fabricius were collected in OCT, sectioned and immunofluorescent assay was performed for the quantification of IgM + B cells, CD4+ cells and CD8α+ cells. The quantitative data from the immunofluorescent assay for IgM+ B cells in spleen (a) for CD4+ cells in lungs (b), bursa of Fabricius (c) and spleen (d) and for CD8α+ cells in lungs (e) are shown. The one-way ANOVA test with Bonferroni's post test for selected comparison was performed to identify group differences and the differences were considered significant at P< 0.05. The bars represent mean ± SEM.

Since we observed that *in ovo* CpG DNA increases KUL01+ cell numbers in lungs, trachea, duodenum, large intestine and bursa of Fabricius post-hatch as an indication of enhanced innate immune response, then we investigated the potential recruitment of cells of the adaptive immune system post-hatch following *in ovo* CpG DNA delivery. On the contrary to the KUL01+ cells response following *in ovo* CpG DNA delivery, IgM + B cells were quantifiable only in lungs and spleen of the examined tissues post-hatch (Table 1). We found that *in ovo* delivered CpG DNA at ED18 increased the number of IgM + B cells in spleen (P<0.05) when compared to both control groups but we could not observe a difference in IgM + B cells in lungs of *in ovo* CpG DNA and PBS or non-CpG DNA delivered groups (P>0.05) (Fig 3a).

Again, on the contrary to the KUL01+ cell response following *in ovo* CpG DNA delivery, CD4+ cells were quantifiable only in lungs, spleen and bursa of Fabricius of the examined tissues post-hatch (Table 1). We found that *in ovo* delivered CpG DNA at ED18 increased the number of CD4+ cells in lungs (P<0.05, Fig 3b), bursa of Fabricius (P<0.05, Fig 3c) and spleen (P<0.05, Fig 3d) of *in ovo* CpG DNA delivered group when compared to PBS or non-CpG DNA delivered controls.

The CD8α+ cells were quantifiable only in lungs, large intestine and bursa of Fabricius of the examined tissues post-hatch (Table 1). We found that *in ovo* delivered CpG DNA at ED18 increased the number of CD8α+ cells in lungs when compared to the *in ovo* non-CpG DNA delivered control group (P<0.05, Fig 3e). However, we could not observe a difference in CD8α+ cells in large intestine (P>0.05) and bursa of Fabricius (P>0.05) of *in ovo* CpG DNA and PBS or non-CpG DNA delivered groups.

### *In ovo* delivered CpG DNA induces protective host response against ILTV infection encountered post-hatch

**Fig 4:**
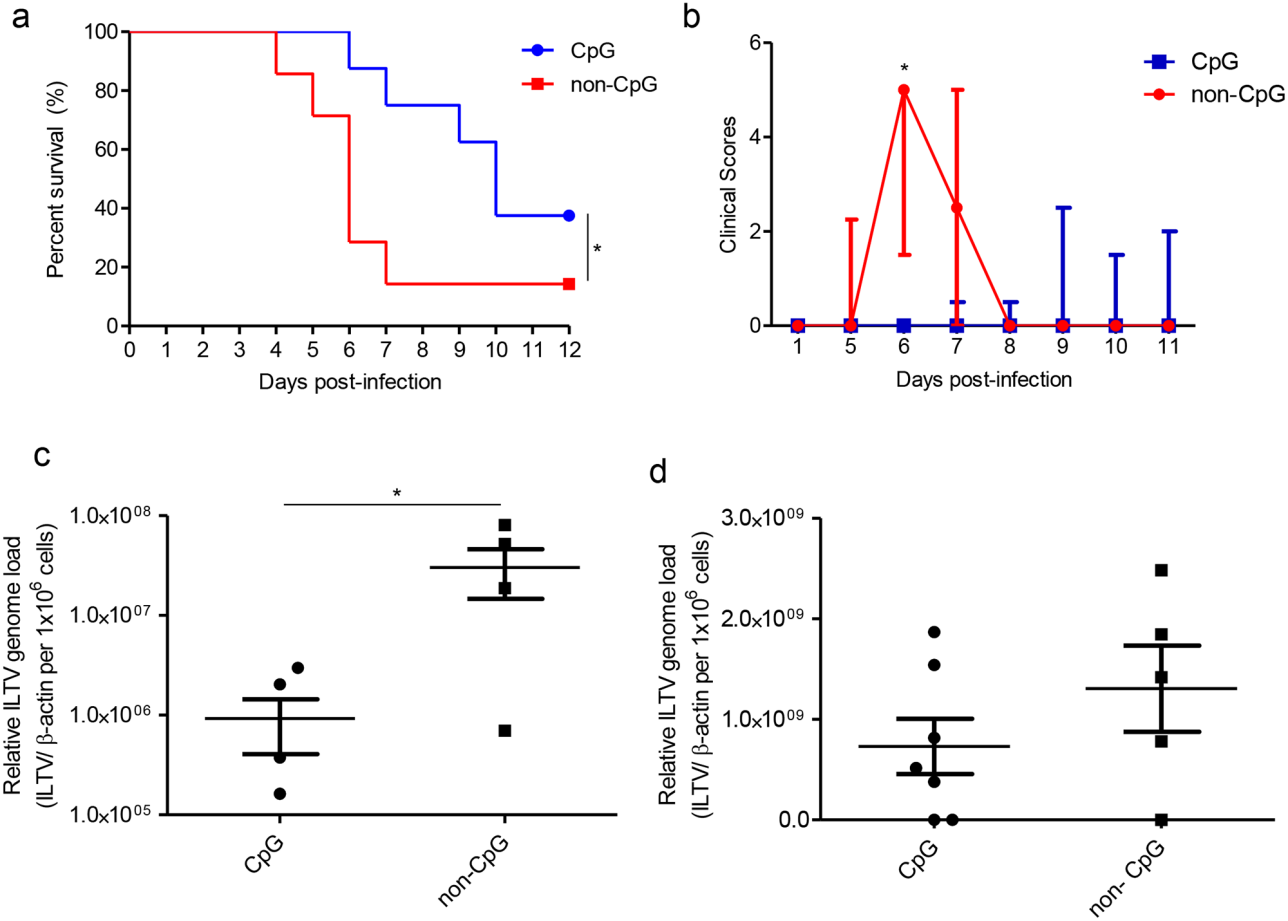
*In ovo* delivered CpG DNA induces protective host response against ILTV infection encountered post-hatch. SPF ED18 eggs were delivered with CpG DNA (n = 8) or non-CpG DNA (n = 7) *in ovo*, the eggs were incubated to hatch and infected with ILTV intra-tracheally at day 1 post-hatch. The chickens were observed for 12 days. At 4 days post-infection, oropharyngeal and cloacal swabs were collected and ILTV genome loads quantified. a) Survival percentage, b) clinical scores, c) ILTV genome loads in cloacal swabs and d) ILTV genome loads in oropharyngeal swabs. Log-rank test was used to identify differences in survival percentages, Mann-Whitney U test was used to identify differences in clinical score data and student's t-test was used to identify differences in ILTV genome loads. The differences were considered significant at P< 0.05. The bars represent median ± inter quartile range in Fig. 4b and mean ± SEM in Fig. 4a, c and d.

Since we observed that *in ovo* delivery of CpG DNA increases recruitments of KUL01+, IgM+ B cells, CD4+ and CD8α+ cells day 1 post-hatch in lungs, trachea, duodenum, large intestine, spleen and bursa of Fabricius, we then investigated to determine if CpG DNA administration influences disease presentation and viral replication after ILTV inoculation at day 1 post-hatch. We found that *in ovo* delivered CpG DNA at ED18 significantly reduced; 1) mortality (P≤0.05, Fig 4a), 2) clinical scores at 6 days post-infection (P<0.05, Fig 4b) and 3) cloacal excretion of the ILTV genome (P<0.05, Fig 4c) but not the oropharyngeal excretion of the ILTV genome (P>0.05, Fig 4d) at 4 days post-infection associated with post-hatch ILTV infection.

**Fig 5:**
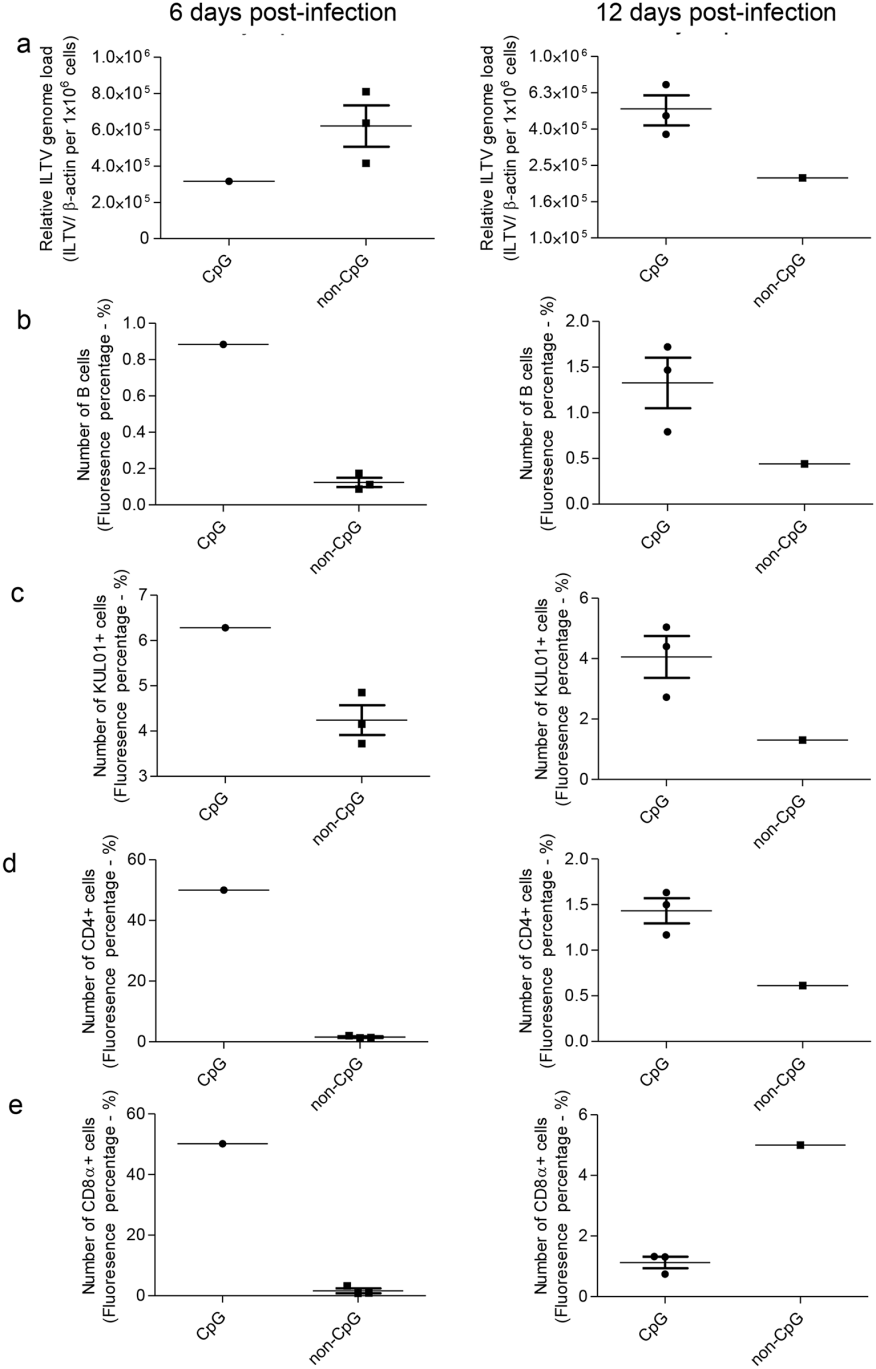
ILTV genome loads and KUL01+, IgM+ B, CD4+ and CD8α+ cell numbers in lungs of *in ovo* CpG DNA and non-CpG DNA treated chickens following ILTV infection post-hatch. The chickens were delivered with either *in ovo* CpG DNA or non-CpG DNA and infected with ILTV intra-tracheally at day 1 post-hatch. The chickens were observed for end points. The lungs were collected for determination of ILTV genome loads and cellular responses from chickens that reached humane endpoint at 6 days post-infection and experimental end point at 12 days post-infection. The ILTV genome loads are given separately for 6 and 12 days post-infection (a). The quantitative data of the immunofluorescent assays done for IgM+ B cells (b), KUL01+ (c), CD4+ cells (d) and CD8α+ cells (e) are shown separately for 6 and 12 days post-infection. Statistical analysis was precluded due to low numbers of animals per group per time point. The bars represent mean ± SEM.

The chickens that reached the selected humane and experimental end points were evaluated for their ILTV genome loads in lungs. Although there is a trend of increased ILTV genome load in lungs at 6 days post-infection in *in ovo* non-CpG DNA treated lungs and at 12 days post-infection in *in ovo* CpG DNA treated liungs (Fig 5a). On the other hand, these end point lungs of ILTV infected *in ovo* CpG DNA delivered group had higher trend of number of cells involve in innate immune responses, IgM+ B and CD4+ cells in both time points (Fig 5b–5d) and CD8α+ cells in 6dpi (Fig 5e) when compared to the lungs of chickens that were ILTV infected and *in ovo* non-CpG DNA delivered. However, statistical analysis of these data were precluded due to less number of samples per group in each time point.

## Discussion

The investigations described here led to three major findings. First, *in ovo* delivered CpG DNA is capable of eliciting significant cellular responses characterized by innate (KUL01+ cells) and adaptive (IgM+ B cells and CD4+ and CD8α+ cells) immune cells day 1 post-hatch. Second, the cellular responses stimulated by *in ovo* delivered CpG DNA in post-hatch chickens are observable in multiple body systems, i.e. lungs, trachea, duodenum, large intestine, spleen and bursa of Fabricius at variable extents (Table 1). Third, the cellular responses induced by *in ovo* delivered CpG DNA were significant at the time of infection (day 1 post-hatch). Whether these innate and adaptive immune cells played a role in the reduction of mortality and virus replication requires further investigation.

For the infection of ILTV we used intra-tracheal infection [23, 30]. When we infected the chickens *via* this route, we observed significantly higher survival of the chickens that received *in ovo* CpG DNA. Although protection against many viral infections depend on antibody-mediated and cell-mediated immune responses, the involvement of antibodies in the protection against ILTV infection is questionable [31, 32] and cell-mediated immune response may play a dominant role. In this context, induction of cellular immune response characterized by KUL01+, CD4+ and CD8α+ cell subsets following *in ovo* delivered CpG DNA may potentially applicable for the control of ILTV infection. Although, we did not investigate the origin of these innate and adaptive immune cells in the examined tissues as to circulating pool of cells or proliferating cells, the evidence of perivascular cuffs was lacking. Following *in ovo* CpG DNA delivery the observed cells were distributed in mucosa or submucosal areas in trachea, duodenum and large intestine. In the lungs, bursa of Fabricius and spleen these immune cells were localizing in the air exchange areas, between bursal follicles or spleen parenchyma respectively following *in ovo* CpG DNA delivery.

Although KUL01+ cells were well distributed in all the examined tissues, CD4+, CD8α+ and B cells were not quantifiable in trachea, duodenum and large intestine (except CD8α+ cells in large intestine) using immunofluorescent assays (Table 1). The lack of CD4+ and CD8α+ cells in trachea, duodenum and large intestine may be due to the lack of colonization of these cells by day 1 post-hatch. It is well known that the colonization of peripheral tissues by CD4+ and CD8α+ cells mostly occur during early post-hatch period [33–35]. However, it has been reported previously that the chickens aged day 1 have much lower numbers of CD4+ and CD8α+ cells when compared to day 7 chickens [35]. The lack of IgM+ B cells in trachea, duodenum and large intestine on day 1 post-hatch may also be related to the lack of B cell colonization in these tissues. This is in agreement with the previous data that the chickens are capable of producing antibodies to orally administered antigen, bovine serum albumin, only after day 8 post-hatch [36]. However, Bar-Shira et al (2003) recorded that mRNA of the CD3γδ and B cell marker (Bu-1) is quantifiable in the various components of the gastrointestinal tract on day 1 post-hatch at a very low quantity when compared to day 4, 6, 8 and 12. This contrasts with our observation that B cells, CD4+ cells and CD8α+ cells are not detectable in duodenum and large intestine. This discrepancy in observations between ours and Bar-Shira et al’s could be explainable by the lower sensitivity of the immunofluorescent technique than real-time PCR technique as well as the fact that not all detected transcripts are translated into functional proteins [37]. Adaptive immune cell recruitment in response to activation of other avian TLR pathways are scarce. However, in other animal models, for example in primates, Wille-Reece et al demonstrated that TLR7/8 ligand induces antigen specific CD8α+ cell response [38]. Similarly, Pasare and Medzhitov demonstrated that the activation of TLR4 signaling by lipopolysaccharide (LPS) influences activation and differentiation of B cells and antigen-specific antibody responses in mice [39].

Avian KUL01+ cells are multifunctional immune cells that play roles producing a number of cytokines, functioning as phagocytic cells and presenting antigens [40]. *In ovo* CpG DNA increases KUL01+ cells in lungs, trachea, duodenum, large intestine and bursa of Fabricius. Previously, we observed that *in ovo* CpG DNA increases expansion of KUL01+ cell numbers in lungs pre-hatch [9, 21, 41] on ED19. One of the limitation of our immunofluorescent technique used to quantify macrophages is that the KUL01 monoclonal antibody detects not only macrophages but also monocytes and interdigitating cells of chicken [42].

IgM+ B cells were higher in spleen in day 1 chickens following *in ovo* delivery of CpG DNA. In agreement with our observation, increases of circulating B cells have been observed in four weeks old chickens when CpG DNA was injected subcutaneously [16]. Although we did not investigate how the IgM+ B cell numbers were increased in spleen of CpG DNA treated chickens, the potential mechanisms may be related to polyclonal activation [11–15] or increased B cell survival due to inhibition of apoptosis [11, 43]. Both these mechanisms are possible since chicken B cells express TLR21 [44].

In agreement with our observation of increased CD4+ and CD8α+ cells in tissues originated from *in ovo* CpG DNA delivered group, increase of circulating and spleen CD3+ and CD4+ cells have been observed in 4 weeks old chickens when CpG DNA is injected subcutaneously [16]. The CD4+ and CD8α+ subsets may be recruited following *in ovo* CpG DNA delivery *via* various mechanisms. First, CpG DNA would have acted as a mitogen increasing these two CD4+ and CD8α+ cell subsets *via* proliferation independent of antigens [45]. Second, it is possible that the increases in CD4+ and CD8α+ cells would have resulted consequent to increased survival of the CD4+ and CD8α+ cells [45]. The direct interactions of CD4+ and CD8α+ cells with CpG DNA are possible since it is known that these CD4+ and CD8α+ cell subsets express receptor for CpG DNA, TLR21 [46, 47]. Third, it is also possible that indirect cytokine mediated activation and proliferation of CD4+ and CD8α+ cells.

The implications of our observations described in the manuscript are many-fold. First, we found that the *in ovo* administration of CpG DNA stimulates the innate and adaptive arms of the immune system as indicated by the expansion of numbers of cells involve in innate immune responses, IgM+ B cell, CD4+ and CD8α+ cells. Whether this induction of innate and adaptive cellular responses leads to the reduced morbidity and mortality of ILTV infection requires further investigations. Second, as one of the major sites of ILTV replication, adaptive immune cell recruitment to the trachea was not significant following *in ovo* delivery of CpG DNA. This was reflected in the oropharyngeal ILTV genome loads which showed only a declining trend in CpG DNA treated chickens when compared to the non-CpG DNA treated chickens. This prompted us to evaluate the ILTV genome loads in the lungs of chickens that reached the end points and we found the same trend of lower ILTV genome loads as observed in oropharyngeal swabs in CpG DNA treated chickens when compared to that received non-CpG DNA. Further analysis indicated the increased trend of KUL01+, IgM+ B, CD4+ and CD8+ cell subsets in CpG DNA treated/ILTV infected chicken lungs at 6 days post-infection when compared to that observed in non-CpG DNA treated/ ILTV infected chickens. Overall, the lack of adaptive immune cell recruitment, particularly CD4+ and CD8α+ cell subsets with higher KUL01+ cell recruitment to trachea by the time of ILTV infection may potentially have contributed to the observed marginal effect of *in ovo* CpG treatment on lung ILTV genome load and oropharyngeal ILTV genome excretion, since it is well documented that cell-mediated rather than antibody-mediated immune response is critical for ILTV protection [31, 32]. Third, the induction of antiviral response pre-hatch *via in ovo* administration is desirable in the field situations in order to provide early host responses at the time of placing the day old chickens in the barn against circulating pathogens in the environment. This protection has been proven against a number of microbial infections in terms of significant reduction in the microbial burdens [23, 48]. *In ovo* route of vaccination is also extensively investigated and has become a commercial practice for the control of many poultry diseases worldwide [49], facilitating the effective and precise delivery of vaccines compared to conventional post-hatch administration methods. Although our findings are preliminary and need further investigations, *in ovo* CpG delivery may provide a basis for developing control measures against ILTV infections in chickens.

In conclusion, we have shown that prophylactic *in ovo* delivery of TLR21 ligand, CpG DNA, leads to increase in KUL01+, IgM+ B cells and CD4+ and CD8α+ cells in lungs, trachea, duodenum, large intestine, spleen and bursa of Fabricius coinciding with the reduction of ILTV infection induced morbidity, mortality and cloacal viral genome load. Although we studied the induction of immune cellular response against only ILTV infection, our observation of induced immune cellular responses in respiratory and intestinal organs following *in ovo* administration of CpG DNA may be potentially effective against other viruses such as infectious bursal disease virus [50, 51] and bacteria such as *E*. *coli* and *Salmonella* species [18–20] infecting in these organs in chicken. Further investigations are required on the functions of these innate and adaptive immune cells in the antiviral response against ILTV.
